# Characterizing the field of Atomic Layer Deposition: Authors, topics, and collaborations

**DOI:** 10.1371/journal.pone.0189137

**Published:** 2018-01-10

**Authors:** Elsa Alvaro, Angel Yanguas-Gil

**Affiliations:** 1 Northwestern University Libraries, Northwestern University, Evanston, Illinois, United States of America; 2 Energy Systems Division, Argonne National Laboratory, Lemont, Illinois, United States of America; Institut Català de Paleoecologia Humana i Evolució Social (IPHES), SPAIN

## Abstract

This paper describes how Atomic Layer Deposition (ALD) has evolved over time using a combination of bibliometric, social network, and text analysis. We examined the rate of knowledge production as well as changes in authors, journals, and collaborators, showing a steady growth of ALD research. The study of the collaboration network of ALD scientists over time points out that the ALD research community is becoming larger and more interconnected, with a largest connected component that spans 90% of the authors in 2015. In addition, the evolution of network centrality measures (degree and betweenness centrality) and author productivity revealed the central figures in ALD over time, including new “stars” appearing in the last decade. Finally, the study of the title words in our dataset is consistent with a shift in focus on research topics towards energy applications and nanotechnology.

## Introduction

Atomic Layer Deposition (ALD) is a technique for depositing thin, conformal films with high control over the thickness that relies on the self-limited interaction of gaseous precursors with the growth surface. Developed in the late 1970s by Tuomo Suntola and co-workers in Finland, it was first introduced with the name Atomic Layer Epitaxy (ALE) and with the original aim of enabling thin film electroluminescent flat panel displays [1]. Since then, it has become a key enabler of semiconductor manufacturing, with Intel introducing ALD into their manufacturing line in 2007 [2, 3]. The range of applications has greatly expanded beyond microelectronics, to include areas such as photovoltaics, energy storage, catalysis, and more.

Several reviews have covered the basics, scientific evolution and applications of ALD [1, 3–7]. The historical development of ALD has also been examined. Puurunen described the invention of ALE, focusing on the early years and the precedents in the Russian literature [8]. Parsons and coworkers described the origins and development of ALD in the last decades of the twentieth century [9]. They also chronicled the growth of the ALD community within the American Vacuum Society (AVS), including the development of the annual International AVS ALD Conference. However, the history of ALD research from the perspective of its scholarly outputs (papers and journals) and authors, including the evolution of the international ALD community through collaborations, has not been examined. We believe that almost forty years after the first ALD patent [10], the field is ripe for such analysis.

The field of ALD also provides a good example of how research communities are born and developed around a specific topic, with a rich publication record that can be explored using bibliometric and social network analysis. Bibliometrics offers a powerful set of tools for studying the outputs of science, and the structure and dynamics of scientific disciplines [11]. Common analyses include examining authors characteristics (such as productivity, collaboration, aggregates by country or institution), papers (such as number or citations), journals (including interdisciplinarity), and statistical aspects of language (analysis of title words, keywords, or abstracts to track the evolution of a field). The use of bibliometric indicators, such as the number of publications and citations, is also common in research evaluation [12].

The combination of bibliometric methods and social network analysis can also provide deep insight on the collaborations within a research field. Co-authorship is a common indicator for collaboration. Co-authorship patterns have been studied from a bibliometric perspective at the micro (publication), meso (institution), and macro (country) levels, showing in general an increase in collaboration in science [13–15]. Network studies have focused on the structure and mechanisms of growth of these networks [16].

Our study seeks to understand how ALD research has evolved over time. In this work, we focus on three different aspects: 1) the evolution of the field from its inception from a bibliometric perspective, including both producers (authors) and outputs (papers and journals); 2) the development of the ALD collaboration network; and 3) changes in research interests and applications, as determined by the evolution of terms in publication titles.

## Materials and methods

### Data collection

The data in this study were extracted from Science Citation Index Expanded (SCIE) using Web of Science. ALD literature was identified using the following queries: “atomic layer deposit*”, “atomic layer epitax*”, or “molecular layer epitax*”, which are the different names that this technique has received since its inception. When extracting the data, we considered a wide period of time (1900–2015) to be able to capture early precedents, but the first record found in this dataset dates from 1981. Of the different types of documents indexed in the database (article, proceedings paper, review, meeting abstract, correction, letter, editorial material, note, book chapter, news item, and correction addition), only articles and reviews were considered for the purpose of this study.

The source data obtained from Web of Science in RIS format were parsed using the gris Python package [17] to extract the following variables from each record: title, source, publication year, document type, doi, times cited, reprint author, reprint country, author, author affiliation, and JCR category. Developed by Research Information Systems, RIS is a tagged format for expressing bibliographic information. The data were stored in a tabular format and further processed and mined using the programming language R [18]. A total of 11288 papers involving 21518 unique author names and 700 journals were included in our dataset. In this work, we have not addressed author name ambiguity: we consider that each unique author name represents a single author.

### Collaboration network

We used our dataset to construct non-directed collaboration networks, where each author is a different node, and two nodes are connected by an edge if two authors have coauthored at least one publication within the timeframe of interest. The network analysis and visualization software Gephi [19] was utilized for reading the network files and doing the analysis.

We focused on two network centrality measures to describe the evolution of the ALD community: degree centrality and betweenness centrality. Degree centrality measures the number of links a node has with other nodes in the network. In our case, it represents the number of collaborators of a scientist in the ALD network; one can think that nodes with higher degree have collaborated with more authors and hence may have more influence in the network. It is worth mentioning that it is also possible that a very high degree may result from a single paper with a very high number of authors. Degree centrality can be calculated from the degree *d(n*_*i*_*)* of each node *n*_*i*_ in the network:

$$C_{D}(n_{i})=d(n_{i})$$ (1)

Betweenness centrality is based on the number of shortest paths passing through a node. Nodes with high betweenness centrality typically play the role of connecting different groups within a network. The betweenness centrality for node *i* can be formulated as follows:

$$C_{B}(n_{i})=\sum_{j,k\neq i}\frac{g_{jik}}{g_{jk}}$$ (2)

where *g*_*jk*_ is the geodesic distance (shortest path) between nodes *j* and *k*, and *g*_*jik*_ is all the geodesics linking nodes j and k that pass through node *i*. It is clear from this expression that a node that is within the shortest path of many different pairs of nodes has a high betweenness centrality.

In this work, we have studied how the ALD coauthorship network evolves over time, including changes in the average path length of the giant component. The average path length is the average of the shortest paths between all pairs of nodes. Average path lengths can only be calculated for node pairs that are in the same network component.

### Analysis of title words

We looked at the frequency with which a selection of terms appeared in the titles of the articles in the dataset. Analysis of title words over time is a common way of determining the evolution and relative importance of research topics [20, 21]. The selection of terms was based on our knowledge of the field, and its applications and history. The analysis of title words was performed using R with the aid of the R package tidytext [22]. A few additional steps were carried out to make sure we were not excluding any terms of scientific relevance:

1. The list of stop words and nonspecific words from the package tidytext was reviewed and modified. Several words that may have chemical meaning (such as symbols of chemical elements) or may be important to the context of this study were excluded from the list. Some examples include: *area*, *order*, *sub*, *well*, *O*, *P*, and *Nd*.
2. Once the list of words was obtained, stemming was done using R scripts to prevent errors in dealing with scientific terms. Additionally, those scripts consolidated terms with the same scientific meaning, such as *Al*_*2*_*O*_*3*_, *alumina*, and *aluminum oxide*, which are different ways of representing the same chemical substance. A list of the consolidated terms can be found in the Supporting Information (S1 Table).

## Results and discussion

### Papers and journals

**Fig 1:**
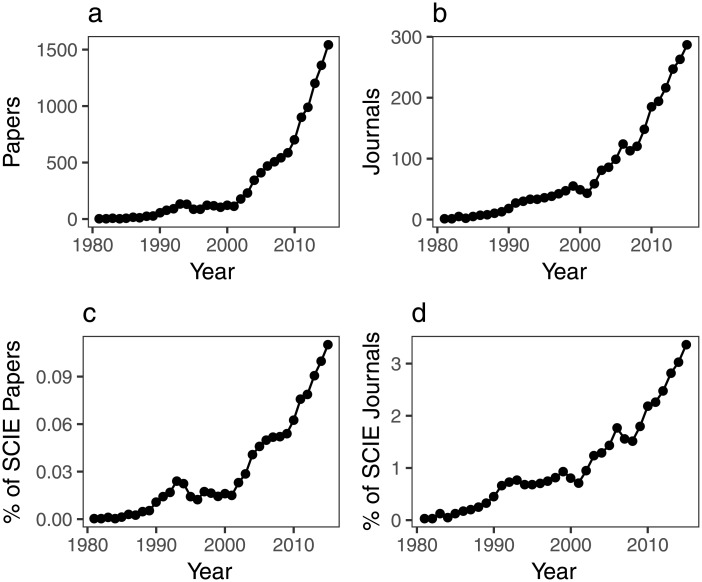
Number of papers (a) and journals (b) that publish ALD research, and percentage among SCIE papers (c) and journals (d).

The first article in our dataset was published in 1981 in *Physica Status Solidi A*, by Tanninen and Oikkonen [23]. Since then, ALD research has substantially grown both in terms of the number of papers and the range of journals that publish ALD research (Fig 1a and 1b). As shown in Fig 1c and 1d, it has also comprised a larger percentage of SCIE papers and journals over time. In all four cases, there is a marked positive increase of the slope starting in 2001, a trend that is maintained until the most recent year considered in our dataset.

Despite the increase in the number of journals that have published ALD research, the ALD publishing activity has been concentrated in a small number of journals. Thus, ten journals have published 37% of the papers in our dataset. Those journals appear in Table 1. *Applied Physics Letters* tops the list (917, 8.1% of the papers), followed by *Thin Solid Films* (530, 4.7%), and *Journal of Applied Physics* (451, 4.0%).

**Table 1:** Top 10 journals by the number of ALD papers published.

	Journal	Papers
1	Appl. Phys. Lett.	917
2	Thin Solid Films	530
3	J. Appl. Phys.	451
4	J. Electrochem. Soc.	406
5	Appl. Surf. Sci.	377
6	J. Vac. Sci. Technol. A	370
7	J. Cryst. Growth	304
8	Chemistry of Materials	290
9	J. Phys. Chem. C	280
10	ACS Appl. Mater. Interfaces	225

To study the evolution of where ALD has been published, we have broken our dataset into four periods: 1981–2000, 2001–2005, 2006–2010, and 2011–2015. The first period covers a longer time span because of the comparatively lower number ALD papers during the early stages of the field. The results are shown in Table 2. *Applied Physics Letters* and *Thin Solid Films* are the only journals that rank highly through all the periods considered, with *Applied Physics Letters* leading the list in the last three periods. It is also worth mentioning that since 2011 three new journals have become popular ALD research destinations: *Journal of Vacuum Science & Technology A*, *ACS Applied Materials & Interfaces*, and *Journal of Physical Chemistry C*.

**Table 2:** Top 5 journals by the number of ALD papers published in different time periods.

	Up to 2000			2001–2005		
	Journal	Papers	% Papers^a^	Journal	Papers	% Papers^a^
1	J. Cryst. Growth	156	12.9	Appl. Phys. Lett.	154	12.1
2	Appl. Surf. Sci.	133	11.0	J. Appl. Phys.	82	6.4
3	Appl. Phys. Lett.	115	9.5	Thin Solid Films	72	5.7
4	Thin Solid Films	93	7.7	J. Cryst. Growth	65	5.1
5	J. Appl. Phys.	58	4.8	J. Electrochem. Soc.	62	4.9
	2006–2010			2011–2015		
	Journal	Papers	% Papers^a^	Journal	Papers	% Papers^a^
1	Appl. Phys. Lett.	320	11.4	Appl. Phys. Lett.	328	5.5
2	J. Electrochem. Soc.	192	6.8	J. Vac. Sci. Technol. A	298	5.0
3	J. Appl. Phys.	138	4.9	Thin Solid Films	228	3.8
4	Thin Solid Films	137	4.9	ACS Appl. Mater. Interfaces	213	3.6
5	Electrochem. Solid State Lett.	109	3.9	J. Phys. Chem. C	192	3.2

^a^ %Percentage over the corresponding time period considered

If we map all journals into the Journal Citation Reports (JCR) categories, we find that ALD research has been published predominantly in journals belonging to the categories *Physics*, *Applied* and *Materials Science*, *Multidisciplinary* (Table 3). In the last five years, *Nanoscience & Nanotechnology* journals have also consistently published ALD research.

**Table 3:** Top 5 JCR categories by number of papers.

	Up to 2000			2001–2005		
	Category	Papers	% Papers^a^	Category	Papers	% Papers^a^
1	Physics, Applied	783	64.5	Physics, Applied	654	51.4
2	Materials Science, Multidisciplinary	395	32.5	Materials Science, Multidisciplinary	415	32.6
3	Physics, Condensed Matter	365	30.1	Physics, Condensed Matter	306	24.1
4	Materials Science, Coatings & Films	316	26.0	Materials Science, Coatings & Films	236	18.6
5	Chemistry, Physical	252	20.8	Chemistry, Physical	222	17.4
	2006–2010			2011–2015		
	Category	Papers	% Papers^a^	Category	Papers	% Papers^a^
1	Physics, Applied	1350	48.1	Physics, Applied	2944	49.1
2	Materials Science, Multidisciplinary	990	35.3	Materials Science, Multidisciplinary	2704	45.1
3	Materials Science, Coatings & Films	525	18.7	Nanoscience & Nanotechnology	1423	23.7
4	Chemistry, Physical	515	18.4	Chemistry, Physical	1379	23.0
5	Physics, Condensed Matter	500	17.8	Physics, Condensed Matter	1062	17.7

^a^Percentage over the corresponding time period considered. Note that one journal can be assigned to multiple JCR categories.

### Authors and collaborations

**Fig 2:**
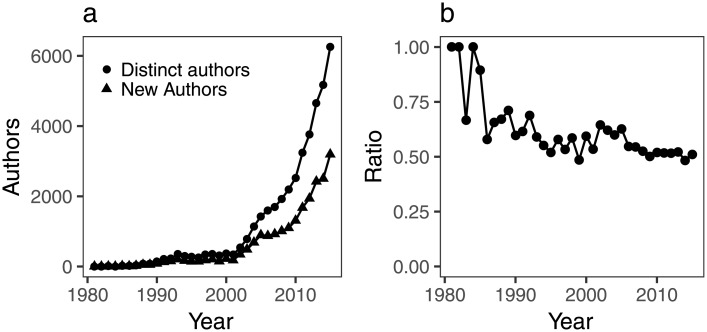
a) Number of distinct authors that publish ALD research, and number of authors that publish ALD research for the first time; b) ration of new and distinct authors.

The number of distinct authors publishing ALD research per year has also increased over time (Fig 2a). This trend has been previously observed in the literature for fields such as Online Laboratory Research [24], and Library and Information Science [25]. Currently, around half of the authors in a given year have not published any ALD paper before. This ratio has reached a plateau, but was higher in the very early years of ALD when the community was very small (Fig 2b).

Of the authors included in the dataset, 10% (2846) have published 5 or more ALD papers. Table 4 shows the most prolific authors overall, and Table 5 shows the most prolific authors through the four periods between 1981 and 2015 considered above. The two most productive authors in this study, *Leskela*, *M* and *Ritala*, *M* (Table 4), are also the only authors who appear consistently highly ranked through all the periods in Table 5. Other scientists with a long, productive ALD focus on their careers are *George*, *SM*, *Hwang*, *CS*, and *Kim*, *H*. In addition, authors who started publishing ALD later and are nowadays highly productive include *Elam*, *JW* and *Kessels*, *WMM*. Finally, some of the most prolific authors of the early stages of ALD (up to 2000) include ALE pioneers like *Bedair*, *SM* and *Nishizawa*, *J* [8].

**Table 4:** Top 10 most productive authors in ALD.

	Author^a^	Papers
1	Leskela, M	329
2	Ritala, M	327
3	Kim, H	240
4	Hwang, CS	199
5	George, SM	198
6	Elam, JW	159
7	Kukli, K	141
8	Kim, J	137
9	Niinisto, L	132
10	Kessels, WMM	115

^a^Author in this table refers to distinct author names

**Table 5:** Top 10 most productive authors in different periods of time.

	Up to 2000			2001–2005	
Author^a^	Papers	% Papers^b^	Author^a^	Papers	% Papers^b^
Leskela, M	91	7.5	Ritala, M	83	6.5
Ritala, M	68	5.6	Leskela, M	76	6.0
Niinisto, L	63	5.2	Kukli, K	47	3.7
Bedair, SM	52	4.3	George, SM	42	3.3
Nishizawa, J	44	3.6	Niinisto, L	40	3.1
Aoyagi, Y	35	2.9	Hwang, CS	39	3.1
Ozeki, M	31	2.6	Aarik, J	38	3.0
Ohtsuka, N	27	2.2	Sajavaara, T	35	2.8
Konagai, M	24	2.0	Lee, JH	31	2.4
Koukito, A	24	2.0	Kim, H	27	2.1
	2006–2010			2011–2015	
Author^a^	Papers	% Papers^b^	Author^a^	Papers	% Papers^b^
Ritala, M	89	3.2	Kim, H	137	2.3
Leskela, M	86	3.1	Elam, JW	92	1.5
Hwang, CS	82	2.9	Ritala, M	87	1.5
Kim, H	76	2.7	Kessels, WMM	79	1.3
George, SM	56	2.0	George, SM	77	1.3
Kim, J	55	2.0	Hwang, CS	77	1.3
Elam, JW	45	1.6	Leskela, M	76	1.3
Fanciulli, M	41	1.5	Kim, DH	68	1.1
Kim, JH	41	1.5	Kim, SH	67	1.1
Jeon, H	40	1.4	Parsons, GN	65	1.1

^a^Author in this table refers to distinct author names.

^b^Percentage of the papers over the period of time considered.

**Fig 3:**
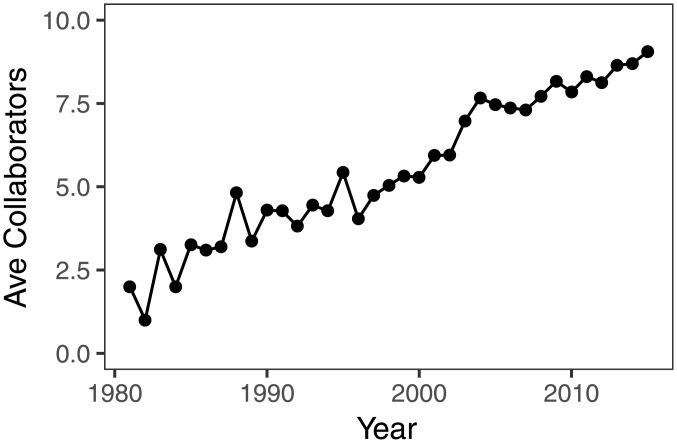
Average number of collaborators per author per year.

ALD is a collaborative field, with only 0.2% of the authors (n = 38) included in the dataset publishing papers as sole authors. The average number of distinct collaborators per author in a given year has steadily increased over time, reaching 9.0 in 2015 (Fig 3). If we also include collaborations occurring in previous years, that is, the cumulative ALD coauthorship network, the average number of collaborators in 2015 rises to 11.8. The average number of collaborators of a scientist is discipline-dependent: different values have been reported for biomedicine (18.1), physics (9.7), and mathematics (3.9) [26], but overall it seems that the average number of collaborators is higher in experimental than in theoretical fields [27].

**Fig 4:**
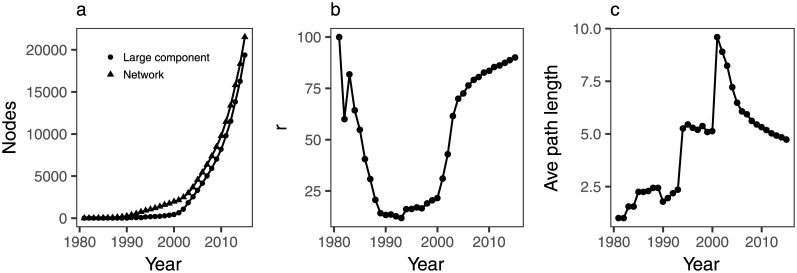
a) Number of nodes of the largest component and the ALD collaboration network; b) relative size of the largest component; c) average path length of the largest component.

In order to study the evolution of collaborations in ALD, we have built cumulative coauthorship networks for the period 1981–2015. A collaboration network may have several isolated clusters of authors who collaborate with each other but not with other authors, or authors who do not collaborate at all. Over time, most collaboration networks develop a large cluster, and this is also the case of the ALD network (Fig 4a). In 2015, 90% of authors belong to the largest connected component (Fig 4b).

**Fig 5:**
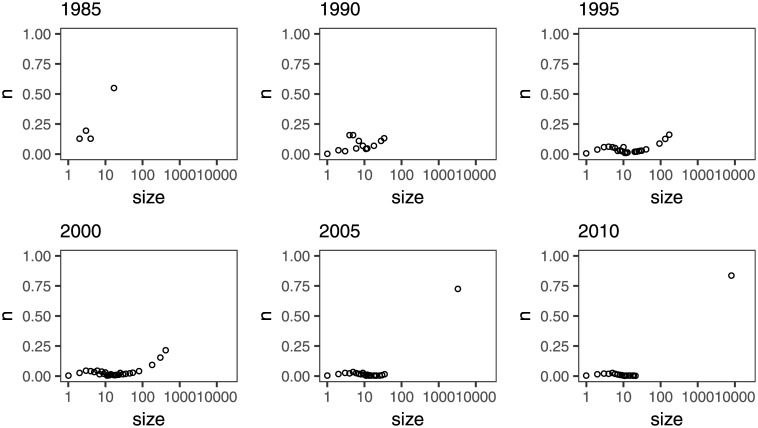
Evolution of component sizes over time.

At the early stages, the number of scientists in the ALD community is very small and most of them belong to the largest connected component (Fig 4b). For instance, the largest component in 1985 spans 55% of the authors (n = 17). As the number of authors grows, the number of isolated clusters also increases and the relative size of the largest component drops, reaching a minimum (12%, n = 88) in 1993. Finally, as separate components merge and new authors join the field, the relative size of the giant component begins to increase. The evolution of component sizes over time is shown in Fig 5.

Around 2001, the relative size of the largest component starts a steady growth. At the same time, the average path length of the largest component, that is, the average distance between all pairs of nodes, begins a declining trend (Fig 4c). This is consistent with the network becoming more interwoven as collaborations start flourishing. This increase in collaborations coincides with the start of the International Annual AVS ALD Conference in 2001 [7]. By 2015, the average path length in the giant component has decreased to 4.7. Given that the ALD network consists of 90% of its largest component, it is a good approximation to infer that it takes, on average, 4.7 steps to get from one scientist to another in the ALD network.

**Fig 6:**
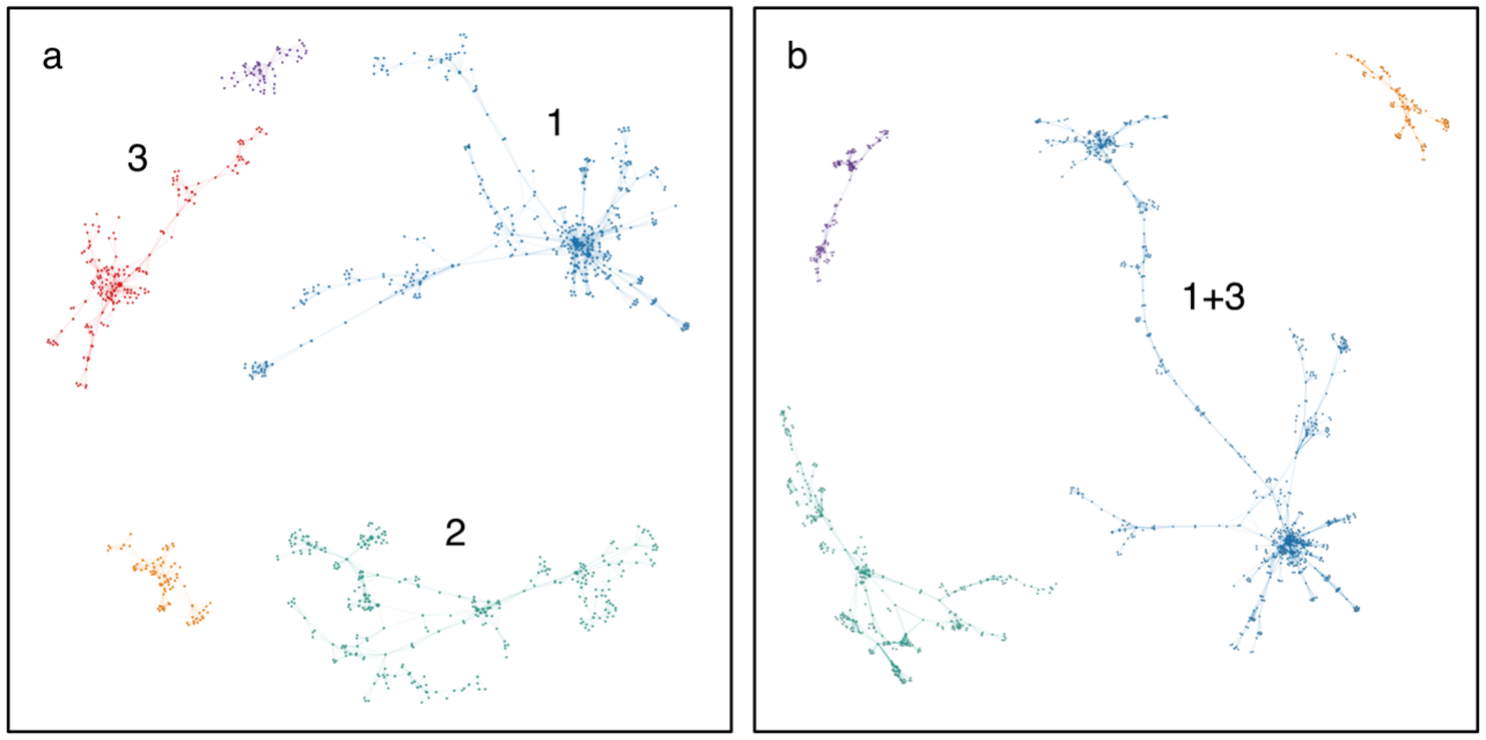
Visualization of the top components in the ALD collaboration network by number of nodes (authors) in 2000 (a) and 2001 (b). Components 1 and 3 in merge, resulting in the increase in the average path length in 2001 observed in Fig 4c.

The evolution of the average path length with time represented in Fig 4c shows two pronounced jumps: one between 1993 and 1994, and a second one between 2000 and 2001. The first jump is consistent with two isolated clusters merging to form a new largest connected component. The second change correlates with the connection of a cluster to the giant component. Similar jumps in the average path length have been previously reported [28]. However, in the case of Lee and coworkers, the intermittent jumps observed were not as pronounced as the ones in this study. The average path length in the ALD network giant component almost doubles in 2001 (from 5.1 in 2000, to 9.6 in 2001). The bridge between the two merging components can be traced to a single paper [29], thus causing such a sudden increase in the mean distance between nodes as authors from two different clusters are connected through a single node (Fig 6).

As collaboration networks evolve, the centrality of authors and collaboration patterns may change. We have applied two measures of network centrality to determine centrality in the ALD research community: degree and betweenness centrality. Degree is the number of nodes connected to a specific node, that is, the number of collaborators of an author. Betweenness centrality is related to the ability of a node to connect between different parts of the network; authors with high betweenness centrality have “more opportunities to broker the flow of information” [30].

As of 2015, *Kim*, *H* is the author with the highest number of collaborators (Table 6), and the highest betweenness centrality (Table 7). Note that this is not necessarily the case: some authors with a high number of collaborators can have a lower betweenness centrality. Also, these measures can also change over time: some authors may be on the rise, and others may be declining because of retiring or focusing their interest in a different field [30]. When we look at how these measures have evolved over the four time intervals considered in this work, we find that two scientists have consistently ranked highly on both centrality measures: *Leskela*, *M* and *Ritala*, *M*. This points out that these authors have been collaborating highly (degree centrality) and diversely (betweenness centrality) since the early days of ALD. Other authors, like *Kim*, *H*, *Kim*, *J*, *George*, *SM*, and *Elam*, *JW*, joined the ALD community later, but have exhibited high degree and betweenness centrality since they joined.

**Table 6:** Top 10 authors based on degree centrality.

	1981–2000				1981–2005		
	Author^a^	Degree	Normalized degree^b^		Author^a^	Degree	Normalized degree^b^
1	Leskela, M	98	0.050	1	Leskela, M	170	0.037
2	Niinisto, L	94	0.048	2	Ritala, M	143	0.031
3	Bedair, SM	79	0.040	3	Niinisto, L	134	0.029
4	Ritala, M	69	0.035	4	Lee, JH	101	0.022
5	Yao, T	37	0.019	5	Kukli, K	87	0.019
6	Elmasry, NA	35	0.018	6	George, SM	80	0.018
7	Nikanen, E	34	0.017	7	Bedair, SM	79	0.017
8	Aoyagi, Y	32	0.016	8	Hwang, CS	77	0.017
9	Karam, NH	30	0.015	9	Kim, H	73	0.016
9	Lappalainen, R	30	0.015	10	Kim, Y	70	0.015
	1981–2010				1981–2015		
	Author^a^	Degree	Normalized degree^b^		Author^a^	Degree	Normalized degree^b^
1	Leskela, M	290	0.030	1	Kim, H	478	0.022
2	Ritala, M	255	0.026	2	Leskela, M	441	0.020
3	Lee, JH	228	0.023	3	Ritala, M	409	0.019
4	Kim, J	211	0.022	4	Elam, JW	381	0.018
5	Kim, H	210	0.021	5	Kim, J	372	0.017
6	George, SM	186	0.019	5	Lee, JH	361	0.017
7	Niinisto, L	179	0.018	7	George, SM	353	0.016
8	Hwang, CS	177	0.018	8	Lee, J	292	0.014
8	Delabie, A	169	0.017	9	Hwang, CS	291	0.014
10	Conard, T	163	0.017	10	Kim, SH	283	0.013

^a^Author in this table refers to distinct author names.

^b^Normalized degree is the degree divided by *n − 1*, being *n* the number of nodes in the network [31].

**Table 7:** Top 10 authors^a^ based on betweeness centrality.

	Up to 2000	1981–2005	1981–2010	1981–2015
1	Leskela, M	Leskela, M	Kim, H	Kim, H
2	Niinisto, L	Kim, H	Lee, JH	Leskela, M
3	Skarp, J	Haukka, S	Leskela, M	George, SM
4	Suzuki, T	Yao, T	Kim, J	Elam, JW
5	Aoyagi, Y	Kim, HS	Ritala, M	Ritala, M
6	Suntola, T	Ritala, M	Hwang, CS	Kim, J
7	Saarilahti, J	Gilmer, DC	George, SM	Kim, S
8	Tammenmaa, M	Lee, JS	Elam, JW	Kessels, WMM
9	Otsuka, N	Cho, M	Lu, J	Hwang, CS
10	Aholpelto, J	Grant, JM	Yasuda, T	Lu, J

^a^Authors in this table refers to distinct author names

### Topics

It is likely that research in ALD has experienced changes in emphasis. In order to understand the evolution of research trends, we analyzed the words contained in the titles of the papers in our dataset, calculating the proportion of papers containing a given term in a given year. Note that a fixed percentage as a function of time represents a topic whose number of papers grows (or decreases) at the same rate as the dataset.

**Fig 7:**
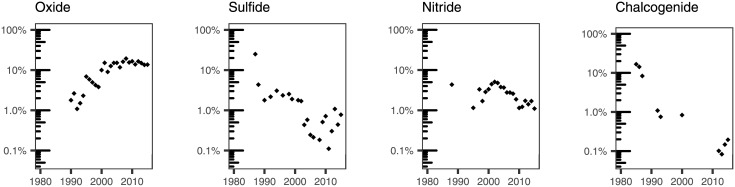
Percentage of ALD papers containing a specific word pertaining to broad types of materials in their titles.

**Fig 8:**
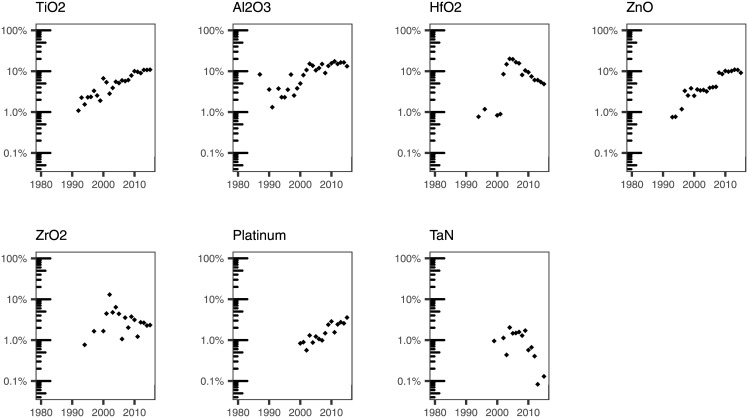
Percentage of ALD papers containing a specific word pertaining to materials that are likely to be grown by ALD in the titles.

**Fig 9:**
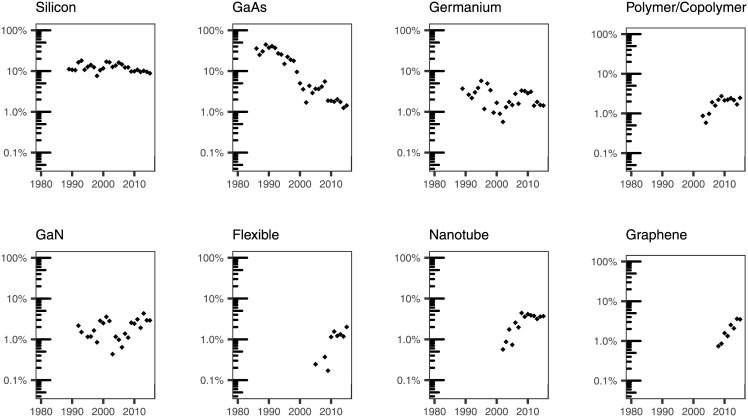
Percentage of ALD papers containing a specific word pertaining to substrate materials in the titles.

**Fig 10:**
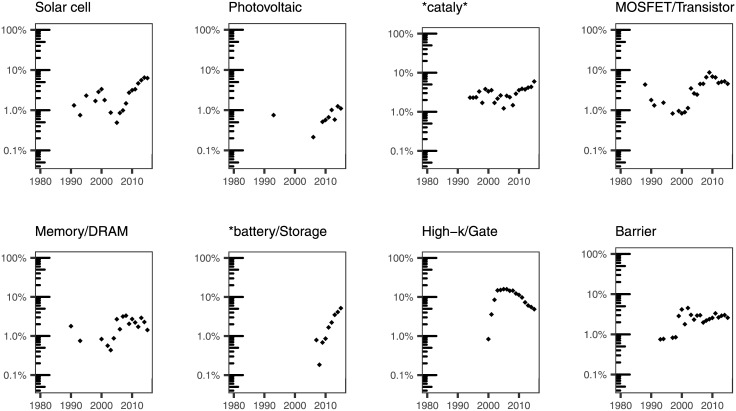
Percentage of ALD papers containing a specific word pertaining to applications of in their titles.

**Fig 11:**
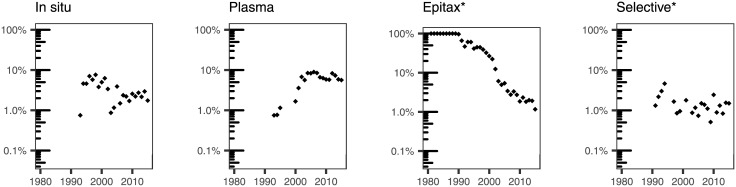
Percentage of ALD papers containing a specific word pertaining to the type of study or process in their titles.

We used our subject expertise to examine specific terms that we grouped as follows: broad categories of materials (Fig 7); specific materials that have been typically grown using ALD (Fig 8); substrates used to deposit materials by ALD (Fig 9); applications of ALD (Fig 10); and terms that have to do with the ALD process (Fig 11).

In the first group, we explored four broad categories of materials: *oxide*, *sulfide*, *nitride*, and *chalcogenide* (Fig 7). Currently appearing in 14% of the ALD paper titles, *oxide* seems to be the most prevalent material type of the four considered, and the only one with a stable neutral trend. *Sulfide*, *nitride* and *chalcogenide* exhibit overall declining trends. In our dataset, the first use of the term *chalcogenide* in a title occurred in 1985. It has appeared in ALD titles only sporadically, and it is now found in 0.2% of the titles. *Sulfide*, which appeared in around 2% of the titles in the 1990s, was present on 0.8% of the titles in 2015. *Nitride* started appearing in 1995, reaching a maximum (5.1%) in 2002, and steadily decreasing later. This is consistent with the interest in nitride materials such as tantalum nitride and titanium nitride as copper diffusion barriers in semiconductor processing.

In addition to these broad categories, we also analyzed a number of specific materials that are grown using ALD. These include: *TiO*_*2*_, *Al*_*2*_*O*_*3*_, *HfO*_*2*_, *ZnO*, *ZrO*_*2*_, *Pt*, and *TaN* (Fig 8). These materials figure prominently in reviews of ALD published in the literature. Of these, *TiO*_*2*_ and *Pt* display upward trends. The first occurrence of *TiO*_*2*_ in our dataset dates back to 1992, and can now be found in around 10% of the titles. In contrast, the first occurrence of *Pt* is comparatively new, taking place in 2000.

Two of the materials explored, *Al*_*2*_*O*_*3*_ and *ZnO*, have reached a plateau after a period of growth. *Al*_*2*_*O*_*3*_ appears in a title in our dataset for the first time in 1987, and has reached a plateau of around 13% since 2000. *ZnO* appeared in 3–4% of the titles from 1999 to 2007, seeing an uptick to 9.2% in 2008, and leveling off to around 10% in 2010. Note that, as relative percentage, these plateau values indicate that the papers mentioning these two materials are growing at the same rate as the field.

The remaining materials studied show a similar behavior characterized by a peak and decline. *ZrO*_*2*_ and *HfO*_*2*_ peaked in 2002 (13% of the titles) and 2004 (20% of the titles), respectively. This behavior is consistent with the exploration of these materials in semiconductor processing, primarily due to their higher dielectric constant [32]. As of 2015, these materials appear in 2.3% (*ZrO*_*2*_) and 4.9% (*HfO*_*2*_) of the titles in our dataset. The decline is even more marked in the case of *TaN*. *TaN* shows a decreasing trend, similarly to what we observed above for nitrides in general. It is also interesting to note that *TaN*’s first occurrence in a title in our dataset happened after the peak of the term *nitride*.

In addition to the materials that are likely to be grown by ALD, we searched for terms that could be attributed to the substrate on which these materials are grown (Fig 9). Much like in the case of materials, the overall trend shows a distinct evolution of words appearing in the title that could be related to substrate materials: while *silicon* has maintained an overall steady presence around 10%, there is a marked decline of *GaAs* of almost two orders of magnitude. *GaAs* occurrence in titles in our dataset has decreased from relatively high numbers in the early days of ALD (at its peak, it appeared in 44% of the titles in 1989) to 1.4% in 2015.

The results in Fig 9 also point out the emergence of new substrate materials: titles mentioning *graphene* have been steadily increasing since 2008, while mentions of *flexible*, *polymer/copolymer*, and *nanotube* start appearing in the early 2000s and level off just before 2010. No apparent trend can be observed in the cases of *GaN* or *germanium*.

Focusing on the evolution of topics related to applications, we also see marked changes over time. In Fig 10, we show a selection of words that can be associated with applications of ALD. These have been chosen taking into account the main application domains described in the literature. One of the most significant results is the emergence of terms that are related to energy applications: the relative weight of words related to energy storage and solar applications has kept increasing in the last years. The earliest mention of *battery/storage takes place in the late 2000s, and its surge correlates with some seminal contributions indicating the ability of ALD films to mitigate capacity fading and enhance the stability of lithium-ion batteries [33, 34]. The trend of words related to solar (*solar cell*, and *photovoltaic*) is less clear, possibly due to the fact that it incorporates different technologies [35], including the passivation of silicon solar cells [36], the development of buffer layers for copper indium gallium selenide (CIGS) solar cells [37, 38], and the application of ALD materials to different flavors of nanostructured photovoltaics, such as dye-sensitized solar cells [39]. In 2015, *solar cell* and *photovoltaic* appear in 6.3% and 1.1% of the titles in our dataset, respectively.

In addition to energy-related terms, we have also considered a set of words that can be associated with semiconductor applications. *MOSFET/transistor* and memory-related terms (*memory/DRAM*) seem to have reached either a plateau or a slightly decreasing trend in the most recent years. Interestingly, the explicit mention of *high-k/dielectric*, which is one of the key properties of some of the most common ALD materials, has a clear peak in the late 2000s. While we have not carried out any studies of co-occurrence of words, this behavior is consistent with the trends observed for *HfO*_*2*_ and *ZrO*_*2*,_ which are two of the materials that were being explored at the time [32]. Finally, there are other applications, such as ALD as barrier or protective coatings and catalysis that seem to have reached a weak dependence with publication year: in the case of catalysis-related words (*cataly*, which includes also terms like *photocalysis* and *electrocatalysis*), their prevalence has always hovered around 1 to 10%, with a small positive slope after 2008. Mentions to *barrier* are found in just 2–3% of the titles in our dataset.

In addition, we have studied words that could provide an indication of the type of study or processes contemplated in the papers in our dataset. These are summarized in Fig 11. The most dramatic change is the decrease of the prevalence of the word epitax*: this correlates with “atomic layer deposition” as a more encompassing substitute of the original “atomic layer epitaxy” for the technique, but it is also an indication that the weight of highly crystalline substrates or growths in our dataset is also very small. Another term where we have observed a substantial evolution is *plasma*. Plasma-assisted ALD relies on a plasma to generate species that are part of the thin film growth process [40]. The fraction of papers in our dataset mentioning *plasma* has reached a plateau of 6–8% of the titles since 2002. Mentions to *in situ* have been decreasing with time from a maximum of 7.6% in 1998 to low single digits. This may reflect a shift of the published papers from the process itself to applications.

**Fig 12:**
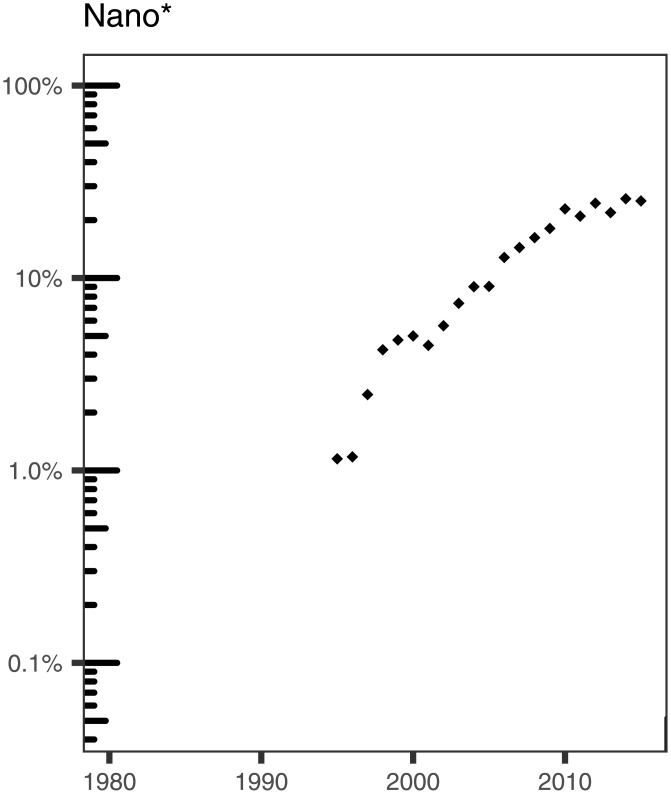
Percentage of ALD papers containing words that start with nano in their titles.

Finally, we examined the occurrence of words starting with *nano* in ALD titles. This can include words like *nanorod*, *nanofiber*, *nanowire*, *nanoparticle*, and more. As can be observed in Fig 12, the first occurrence of nano* in a title in our dataset appears in 1995 and the exact word was *nanostructured* [41]. The occurrence of nano* in titles in our dataset exhibits an overall upward trend, and seems to be currently stabilized around 25% of the titles. The US National Nanotechnology Initiative (NNI) was created in 2000, which may have fostered the exploration of ALD for nanotechnology applications; this may be consistent with the solid growth observed in the last 15 years.

## Discussion and conclusions

In this paper, we have depicted the evolution of ALD research since the 1980s. The earliest papers in our dataset can be traced to a small group of researchers mostly affiliated with Finnish institutions. Since then, more than 11,000 papers have been published by roughly 21,500 scientists. ALD as a research field has grown according to all the metrics considered, including the number of papers, journals, and authors.

The ALD collaboration network has become larger and more interconnected, and currently 90% of the scientists are part of the largest connected component. This number is consistent with the size of the largest connected component reported by Newman for Biology (92%) and Physics (85%) collaboration networks [26]. It is interesting to note that the growth of the giant component up to 2001 takes place through the merging of a number of rather large clusters, as pointed out by the evolution of the network component sizes and the dramatic jumps observed in the average path length. This seems to indicate that ALD research progressed separately in several clusters of scientists that later became connected, and that collaborations grew rapidly after 2001, probably fostered by the annual ALD AVS conference. This interpretation is in agreement with the history of the early days of ALD published by Puurunen [8] and Parsons and coworkers [9].

The analysis of the title words in ALD papers is consistent with an evolution of topics or interests as a function of time. A particularly striking case is the appearance of terms related to nanoscience or nanotechnology, which are absent from our dataset before 1995 and have steadily grown to be featured in around 25% of the titles in 2015. This fact is also in agreement with an increase of the ALD papers published in journals of the JCR category Nanoscience & Nanotechnology in the last five years.

The comparison of the trends observed in title words combined with our subject expertise indicates that some of the changes in topics may be correlated. One example is the trends for the oxides of hafnium and zirconium and high-k/gate dielectrics. This suggests that a more detailed study on the co-occurrence of words could provide further information on the cognitive structure of ALD. This analysis, however, falls outside the scope of this work.

Our view of ALD as a research field is informed by the way in which we have built our dataset: We have used the three terms employed to name the field in different periods of its history. Many different approaches have been described in the literature to define the field of study. Some examples include using keywords (or index terms or thesauri) in literature databases while limiting to specific set of journals [42], building papers around a small number of key authors, analyzing a subject-specific database, such as ArXiv or PubMed, using interactive query formulation [21, 43], or defining a set of core journals [20, 25]. Many of these strategies are not applicable to this study. For instance, there are not journals that publish exclusively ALD research. Besides, as we have observed, ALD draws from several fields, including Materials Science, Applied Physics, and Physical Chemistry, and has been featured in multiple journals, which have fluctuated over time.

By choosing a criterion based on the presence of certain key terms in the bibliographic record, we may be excluding papers in which ALD does not play a prominent role (such as contributions that mention ALD in the experimental section). Likewise, it is also possible that we have false positives, such as papers that include ALD as a keyword but only tangentially deal with it. However, the large size of the giant component is consistent with a low density of false positives in our dataset. An alternative way of defining our dataset could involve studying the papers presented at the International Annual ALD AVS Conference. However, this method would eliminate authors whose contributions predate these conference series, leading to a dataset biased towards post-2001 research. We would be also excluding authors that do not present their research at this conference.

Finally, a key problem in information science is author disambiguation. This is a well-known problem when attributing authorship: 1) A single name may be representing several individuals; and 2) two or more names may be representing the same individual (e.g. Elam JW and Elam J). Author name disambiguation is a complex task that is being actively investigated [44]. Milojevic [45] divides unsupervised methods in: 1) simple or name-based, which use last name and first or all initials; and 2) advanced methods [44]. It is also well known that simple methods introduce misidentification errors that impact the statistical properties of networks. [46, 47]. Taking advantage of our subject knowledge, it would be in principle possible to tackle many of these issues by manually inspecting the more than 21,000 author names in our dataset. This would allow us to establish a comparison between different author disambiguation approaches. However, such a study falls outside the scope of the present work.

## Supporting information

S1 Table (DOCX)
