# Supplementary material for: Characterizing the field of Atomic Layer Deposition: Authors, topics, and collaborations
Source: PLoS One. 2018 Jan 10;13(1):e0189137. doi: 10.1371/journal.pone.0189137 (PMC5761841; doi:10.1371/journal.pone.0189137)
Supplement: S1 Table — (DOCX) [file pone.0189137.s001.docx]

**S1 Table 1. Consolidated words used in the analysis of title words^a^**

| **Term** | **Represents** |
| --- | --- |
| al2o3 | al2o3, alumina, aluminum oxide, aluminium oxide, aluminum oxides, aluminium oxides |
| hfo2 | hfo2, hafnia, hafnium dioxide, hafnium oxide |
| zro2 | zro2, zirconia, zirconium dioxide, zirconium oxide |
| tio2 | tio2, titania, titanium dioxide, titanium oxide |
| platinum | Platinum, pt |
| tan | tan, tantalum nitride, tantalum mononitride |
| zno | zno, zinc oxide |
| silicon | silicon, si |
| gaas | gaas, gallium arsenide |
| germanium | germanium, ge |
| polymer/copolymer | polymer, polymers, polymeric, copolymer, copolymers, copolymeric |
| gan | gan, gallium nitride |
| *cataly* | catalyst, catalysts, catalyst, catalysis, catalyzed, catalytic, catalytically, electrocatalysis, electrocatalysts, electrocatalytics, electrocatalyst, photocatalysis, photocatalysts, photocatalytic, photocatalyst |
| mosfet/transistor | mosfet, mosfets, transistor, transistors |
| memory/dram | memory, memories, dram, drams |
| *battery/storage | battery, batteries, storage, microbattery, microbatteries |
| high-k/gate | high-k, high-kappa, gate |
| epitax* | epitaxial, epitaxially, epitaxy |
| selective* | selective, selectively |
| nano* | words that start with nano |

^a^Plurals of the words analyzed not included on this table are identified during the stemming process. For instance, “nanotube” represents “nanotube” and “nanotubes”, and “plasma” represents “plasma” and “plasmas”.
